# Type 2 diabetes and the risk of synovitis-tenosynovitis: a two-sample Mendelian randomization study

**DOI:** 10.3389/fpubh.2023.1142416

**Published:** 2023-05-04

**Authors:** Jiale Guo, Caiju Peng, Qionghan He, Yehai Li

**Affiliations:** ^1^Department of Orthopedics, Chaohu Hospital of Anhui Medical University, Hefei, China; ^2^Chaohu Hospital of Anhui Medical University, Hefei, China

**Keywords:** diabetes mellitus, synovitis, tenosynovitis, Mendelian randomization analysis, musculoskeletal diseases, risk factors, genome-wide association study

## Abstract

**Introduction:**

It has been shown that people with type 2 diabetes have a higher risk of synovitis and tenosynovitis, but previous studies were mainly observational, which may be biased and does not allow for a cause-and-effect relationship. Therefore, we conducted a two-sample Mendelian randomization (MR) study to investigate the causal relationship.

**Method:**

We obtained data on “type 2 diabetes” and “synovitis, tenosynovitis” from published large-scale genome-wide association studies (GWAS). The data were obtained from the FinnGen consortium and UK Biobank, both from European population samples. We used three methods to perform a two-sample MR analysis and also performed sensitivity analysis.

**Results:**

The results of all three MR methods we used for the analysis illustrated that T2DM increases the risk factor for the development of synovitis and tenosynovitis. Specifically, for the IVW method as the primary analysis outcome, OR = 1.0015 (95% CI, 1.0005 to 1.0026), *P* = 0.0047; for the MR Egger method as the supplementary analysis outcome, OR = 1.0032 (95% CI, 1.0007 to 1.0056), *P* = 0.0161; for the weighted median method, OR = 1.0022 (95% CI, 1.0008 to 1.0037), *p* = 0.0018. In addition, the results of our sensitivity analysis suggest the absence of heterogeneity and pleiotropy in our MR analysis.

**Conclusion:**

In conclusion, the results of our MR analysis suggest that T2DM is an independent risk factor for increased synovitis and tenosynovitis.

## 1. Introduction

Synovitis and tenosynovitis are a group of aseptic inflammatory diseases associated with acute trauma or chronic strain. It is estimated that synovitis and tenosynovitis have a high prevalence in the population and are a common group of musculoskeletal disorders that can seriously affect personal health and work life and impose high healthcare costs on society (1–3). In 2008, the analysis of data on claims for case allowances in Brazil illustrated that the overall prevalence of synovitis and tenosynovitis was 10.9/10,000 and occurred mainly in the physically active population, being the second most common type of all musculoskeletal disorders (after back disorders) (4). If only women are considered, synovitis and tenosynovitis are the most prevalent and persistent chronic diseases. The Connecticut Department of Labor's annual report on the causes of work-related chronic diseases shows that 10% of musculoskeletal disorders are tendinopathies. For workers, the overall prevalence of tenosynovitis was 3.1%; 5.5% in high prevalence occupations; and 2.5% in low prevalence occupations (5). Type 2 diabetes, the most common type of diabetes, accounts for 90% of all diabetes (6, 7). The global prevalence of diabetes is estimated to be 9.3% (463 million people) in 2019, rising to 10.2% (578 million people) by 2030 and 10.9% (700 million people) by 2045 (8). And 50% of these patients do not know they have diabetes. Diabetes has now been shown to be a risk factor for multiple diseases, including cardiovascular disease (9, 10), kidney disease (11), and more. Some studies have shown that people with diabetes have a higher risk of synovitis and tenosynovitis (12–14). However, these studies are mainly observational studies, which are more likely to be influenced by confounding factors. And traditional observational studies can only obtain correlational relationships, not exact causal relationships (15).

Mendelian randomization (MR) is a method that uses genetic variation as an instrumental variable (IV) for exposure to estimate the causal association between exposure and certain outcomes (15–17). MR is conceptually similar to a randomized controlled study because genetic variation is randomly assigned during gamete formation before any confounding factors interfere, and is uniformly distributed across the population (17). Furthermore, alleles are fixed across individuals and do not change with disease onset or progression. Therefore, causal inferences obtained from MR analysis are less susceptible to bias from residual confounders and reverse causality (17–21). And with the increasing abundance of genome-wide association study (GWAS) data published by large consortia, gives MR studies a sufficient sample size to analyze reliable results (22–24). Here we performed a two-sample MR study to assess the effect of T2D on the risk of synovitis and tenosynovitis.

## 2. Method

### 2.1. Study design

**Figure 1:**
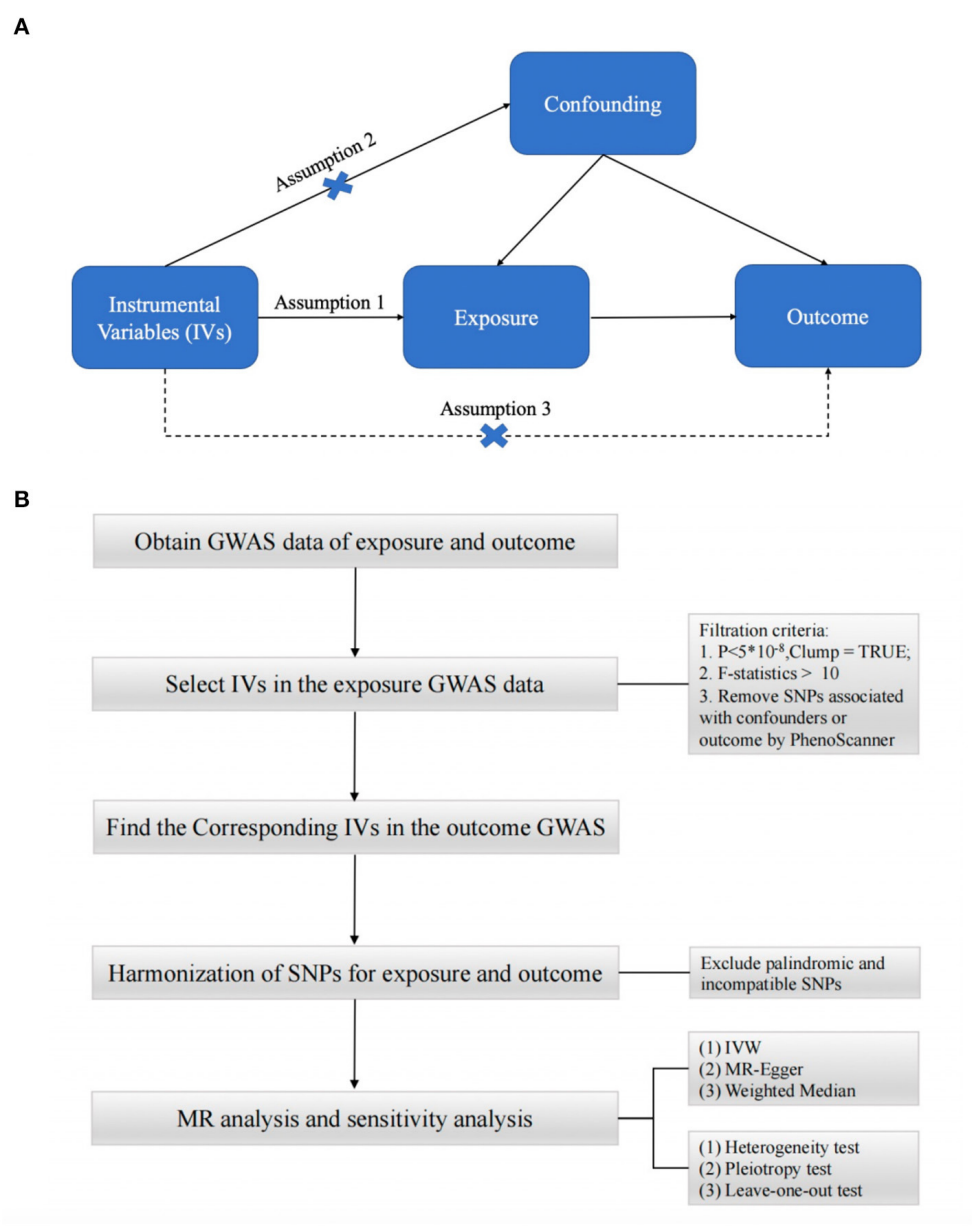
**(A)** Three assumptions of Mendelian randomization. **(B)** Flow chart of Mendelian randomization.

To obtain reliable results, mendelian randomization (MR) studies must be based on three assumptions (15–17) (Figure 1A): (1) IV is strongly correlated with exposure factors; (2) IV is not correlated with any confounding factors affecting exposure and outcome; (3) IV is not directly correlated with outcome, and his effect on outcome is only reflected through exposure (25). In this study, we performed a two-sample MR (26) analysis to explore the causal relationship between type 2 diabetes and synovitis and tenosynovitis. For the two-sample MR study, the association between variance and exposure was estimated in one dataset, and the association between variance and outcome was estimated in the second dataset. Our analysis process consisted of five main parts: (1) reading the exposure factor GWAS data; (2) selecting the appropriate instrumental variables; (3) reading the outcome GWAS data and extracting the SNPs of the aforementioned instrumental variables; (4) preprocessing the exposure factor and outcome GWAS data to make them in a uniform format; and (5) performing MR analysis and sensitivity testing. The flow chart of the whole analysis is shown in Figure 1B.

### 2.2. Data source

Genetic variants (SNPs) associated with type 2 diabetes were extracted from published Genome-Wide Association Study (GWAS) data published by the FinnGen Consortium, using the “Type 2 diabetes” phenotype in this study. The GWAS included 215,654 Finnish subjects, including 32,469 cases and 183,185 controls. The pooled data for tenosynovitis and synovitis were obtained from the GWAS phenotyped “M65 Synovitis and tenosynovitis” published by the UK biobank, which was derived from a European sample of 361,194 subjects, including 2,812 cases and 358,382 controls. Our MR study was conducted using publicly available studies or shared datasets and therefore did not require additional ethical statements or consent.

### 2.3. Selection of IV

For the first assumption, “IV is highly associated with exposure”, we selected SNPs from the European GWAS under the genome-wide significance threshold (*p* < 5 × 10^−8^) associated with exposure interest as potential SNPs. we then used the clump function (r^2^ = 0.001, kb = 10,000) to remove selected single nucleotide linkage disequilibrium (LD) between selected single nucleotide polymorphisms (SNPs). These SNPs were excluded from the subsequent analysis. We used the F-statistic to assess weak instrumental variable effects (27). When the F-statistic is < 10, we consider the genetic variation used to be weak IV, which may have some bias on the results. Then for the second assumption, “IV is not associated with confounding factors”. We further examined whether these SNPs were associated with potential risk factors such as BMI, smoking, and hyperlipidemia by using a comprehensive web-based genotype-phenotype association database “PhenoScanner” (http://www.phenoscanner.medschl.cam.ac.uk). For SNPs associated with confounding factors, we manually performed culling. At the genome-wide significance level (p < 5 × 10^−8^), we removed SNPs associated with these potential confounders. For the third assumption, “IV is not associated with outcome,” we needed to manually remove SNPs associated with outcome (p < 5 × 10^−8^). After extracting the remaining SNPs from the outcome data, we performed harmonization to ensure that the effects of IVs on exposure and outcome corresponded to the same effect alleles while excluding SNPs with palindromic sequences that could not determine the orientation and incompatible SNPs. The last remaining SNPs were used as IVs for the next MR analysis.

### 2.4. MR analysis

To avoid the effect of potential pleiotropy, we used three different MR methods (inverse variance Weighted (28) (IVW), MR-Egger regression (29) and weighted median (30)) to assess the causal effect between T2DM and synovitis and tenosynovitis. The results of the IVW method were used as the main results. In the hypothesis of IVW, we considered that all SNPs were not polyvalent (all were valid IVs). In addition, considering that the results of GWAS were done after standardization for multiple phenotypes, we considered a positive relationship between outcome and exposure. Briefly, the IVW method assumes that all IVs are valid IVs, the weighted median method allows 50% of the IVs to violate the IVs assumption, and MR-Egger allows all IVs to violate the IVs assumption. Furthermore, in MR-Egger's hypothesis framework, we consider the existence of the intercept and use it to assess pleiotropy. If this intercept term is 0, the results of the MR-Egger regression model are very close to IVW; however, if the intercept term is very far from 0, it indicates that these IVs may have horizontal pleiotropy. MR-Egger and Weighted median were used as complements to IVW estimation. These methods, although less efficient (wider CI), can provide reliable estimates under a wider range of conditions.

### 2.5. Sensitivity analysis

To demonstrate the reliability of our results, we performed a sensitivity analysis to detect potential horizontal pleiotropy and heterogeneity in our analysis. Cochran's Q test was used to detect potential heterogeneity. Cochran's Q statistic assessed the heterogeneity between genetic variants and considered heterogeneity when *p* < 0.05. And we plotted funnel plots based on the results. Subsequently, MR-Egger intercept tests were performed to provide estimates of horizontal pleiotropy (*p* < 0.05 was considered as the presence of an intercept and horizontal pleiotropy). MR-PRESSO analysis was performed to further analyze the pleiotropy and to look for sources of pleiotropy (31). A leave-one-out analysis was also performed to assess whether causality was depending on or biased toward any single SNP. All statistical analyses were performed using the “TwoSampleMR” package (https://github.com/MRCIEU/TwoSampleMR) of R software (version 4.1.3).

## 3. Results

### 3.1. Instrumental variables

Through the above process of screening, we finally selected 35 SNPs as IVs for the final analysis. All IVs were performed with an F-statistic > 10, indicating a low probability of weak IV bias. The details information on all the IVs is displayed in Appendix 1.

### 3.2. MR analysis

**Figure 2:**
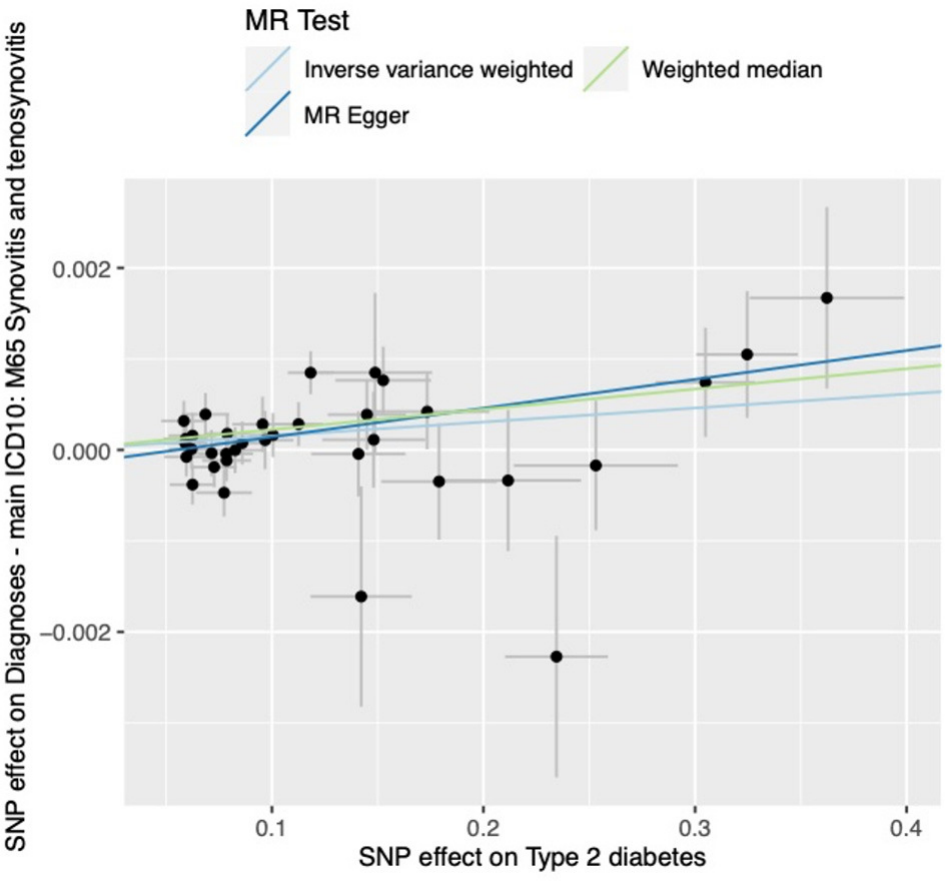
Scatterplot of MR analysis.

**Figure 3:**
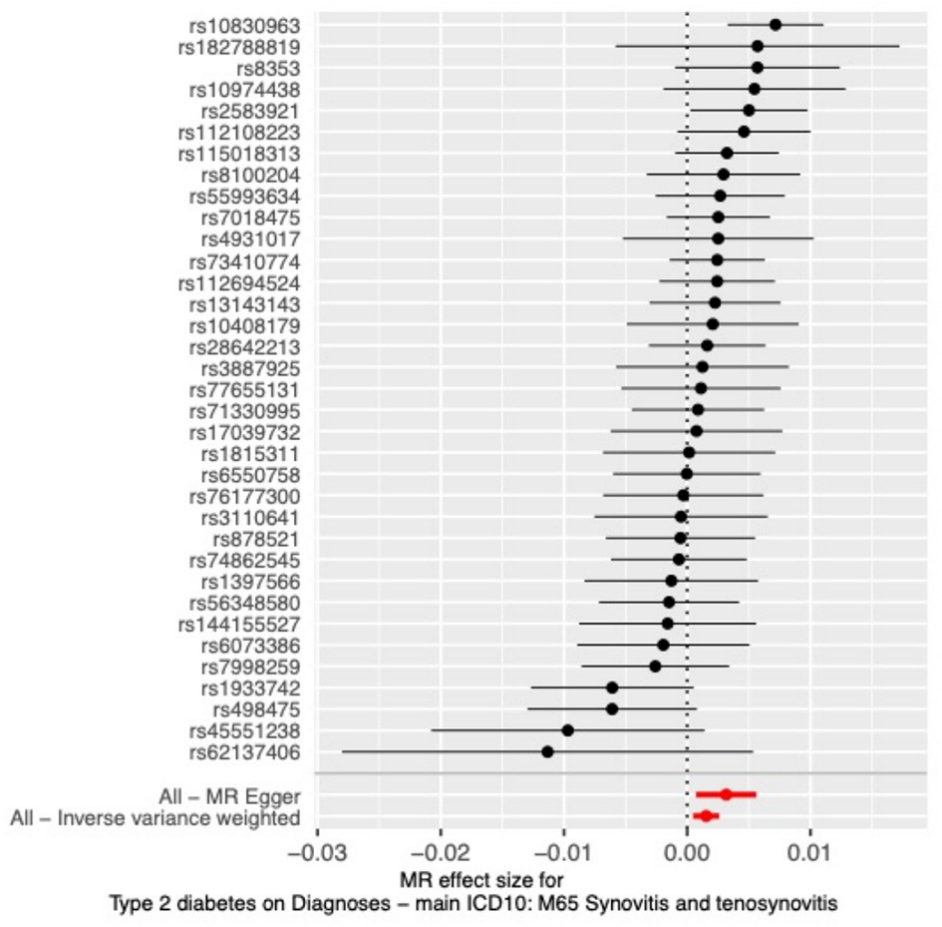
Forest plot of MR analysis.

The results of all three MR methods we used for the analysis illustrated that T2DM increases the risk factor for the development of synovitis and tenosynovitis. Specifically, for the IVW method as the primary analysis outcome, OR = 1.0015 (95% CI, 1.0005 to 1.0026), *P* = 0.0047; for the MR Egger method as the supplementary analysis outcome, OR = 1.0032 (95% CI, 1.0007 to 1.0056), *P* = 0.0161; for the weighted median method, OR = 1.0022 (95% CI, 1.0008 to 1.0037), *p* = 0.0018. In addition, based on the results of the MR analysis, we plotted scatter plots (Figure 2) and forest plots (Figure 3).

### 3.3. Sensitivity analysis

**Figure 4:**
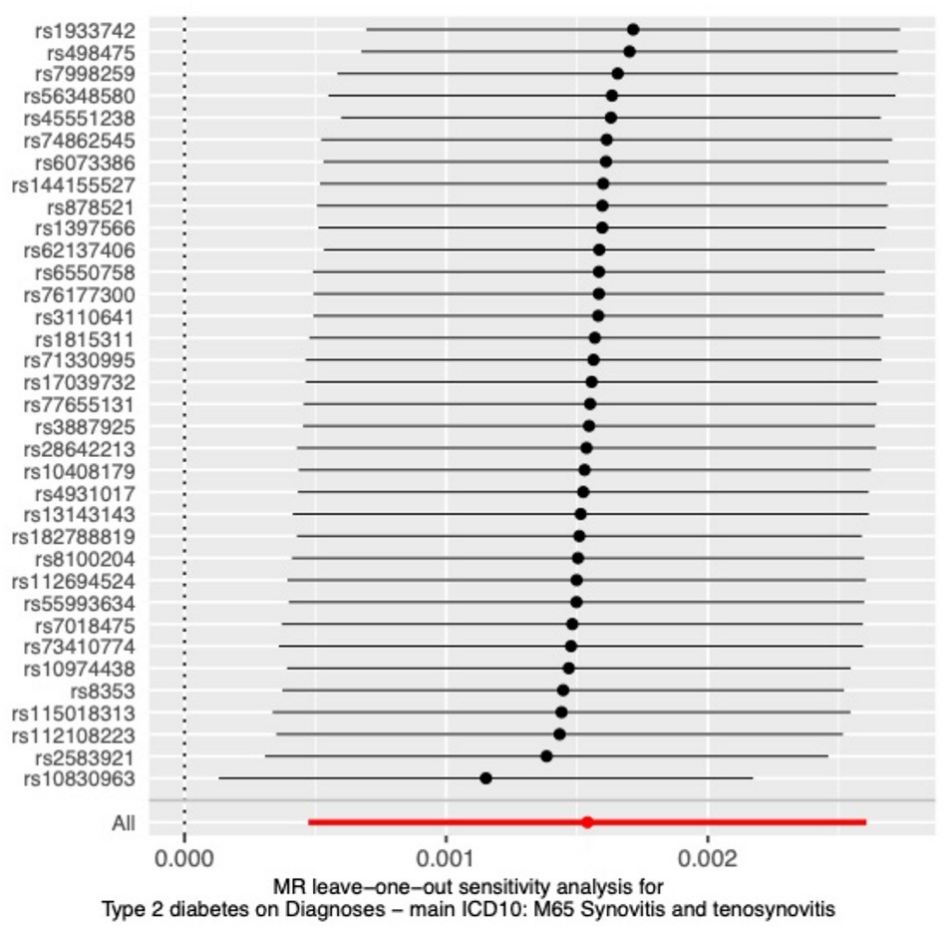
Sensitivity analysis by the leave-one-out method.

To further verify the reliability of the results, we performed a sensitivity analysis to examine the heterogeneity and pleiotropy of MR. The results of Cochran's Q test showed no heterogeneity in IVs (*p* > 0.05), and the funnel plots we plotted are shown in Appendix 2. No significant pleiotropy or SNPs with outliers (*P* > 0.05) were found in the MR-PRESSO analysis. The results of the MR-Egger intercept test also showed no pleiotropy in our analysis (*p* = 0.16). The results of the leave-one-out test showed that causality did not rely on or bias any single SNP (Figure 4).

## 4. Discussion

In contrast to previous observational studies, the MR analysis we performed aimed to investigate the causal relationship between T2DM and the risk of synovitis and tenosynovitis. To our knowledge, this is the first two-sample MR study to examine the causal relationship between T2DM and the risk of synovitis and tenosynovitis using large GWAS data. By MR analysis, we found that T2DM increases the risk of synovitis and tenosynovitis in the population. Moreover, there was no heterogeneity or pleiotropy in our study, and the results of the sensitivity analysis suggest that our results are reliable.

Previous studies are still controversial in stating whether T2DM increases the risk of synovitis and tenosynovitis in the population. Results from an analysis of the Taiwan Health Insurance Claims Database illustrated that diabetes mellitus was significantly associated with the occurrence of stenosing flexor tenosynovitis (SFT) (RR, 1.74; 95% CI 1.54-1.97) (12). Cross-sectional studies from Arabia (32) and Amman, Jordan (33), also indicate a greater probability of tenosynovitis than the general population. These studies show that T2DM increases the risk of synovitis and tenosynovitis in people of other races. Although most studies support that T2DM increases the risk of developing tenosynovitis and synovitis, there are also studies that illustrate that the incidence of tenosynovitis does not differ significantly among patients with T2DM (34). In addition, all of these studies were low on the evidence-based medical evidence scale, with the potential for various serious risks of bias. However, our MR analysis largely avoided confounding factors and had an effect similar to that of a randomized controlled trial. Moreover, the sample size included in the analysis was large, giving sufficient evidence to resolve the controversy. In addition, all data included in our study were derived from the European population, avoiding the bias of population heterogeneity.

There are fewer studies on the effect of diabetes on the risk associated with musculoskeletal disorders. A previous MR demonstrated T2DM as an independent risk factor for carpal tunnel syndrome (35). The results of all three MR methods analyzed in our study indicate that T2DM increases the risk of synovitis and tenosynovitis, and there was no significant heterogeneity or pleiotropy in the results of the analysis. We can use the results of the MR analysis to screen for people at risk in advance. That is, people with diabetes are more likely to develop synovitis and tenosynovitis, and for patients with diabetes we may be able to avoid the development or further progression of synovitis and tenosynovitis through early prevention and screening. In addition, synovitis and tenosynovitis of the hand have previously been suggested in studies as clinical and diagnostic tools for diabetic patients (32). Our findings provide some degree of justification for realizing this possibility.

However, we have some limitations in this study. First, both GWAS datasets we included in our study were derived from European populations, which to some extent limits the generalization of the results to other populations (e.g., Asians and Africans). Therefore, our findings should be used with caution when preparing for application to other populations. Second, our exposure data are for the “M65 Synovitis and tenosynovitis” phenotype, including systemic synovitis and tenosynovitis, without stratification by specific disease type and severity, patient gender, or age. Third, we excluded only SNPs associated with known confounders, such as BMI, blood lipids, and BMI-related characteristics (arm fat mass, arm fat removal, and waist circumference), and other unknown confounders need to be further investigated. Finally, it should be noted that SNPs refer to the biological function of an individual and cannot fully replace the T2DM phenotype. Whereas T2DM is genetically as well as environmentally, lifestyle, and epigenetic modifications, our results can only partially explain the causal effect of T2DM on synovitis and tenosynovitis.

## 5. Conclusion

In conclusion, we performed MR analysis using data from a large sample of GWAS analyses, and the results of our analysis showed that T2DM is an independent risk factor for increased synovitis and tenosynovitis. And the results of our sensitivity analysis proved that the results of our MR analysis are stable and reliable.

## Data availability statement

Publicly available datasets were analyzed in this study. This data can be found here: UK-Biobank: https://www.nealelab.is/uk-biobank; the FinnGen: https://www.finngen.fi/en.

## Author contributions

Conceptualization: YL. Methodology: JG and CP. Writing: JG and QH. Review and editing: CP and QH. All authors contributed to the article and approved the submitted version.
