# Supplementary figures and images for: Type 2 diabetes and the risk of synovitis-tenosynovitis: a two-sample Mendelian randomization study
Source: Front Public Health. 2023 May 4;11:1142416. doi: 10.3389/fpubh.2023.1142416 (PMC10192564; doi:10.3389/fpubh.2023.1142416)

## MR Method

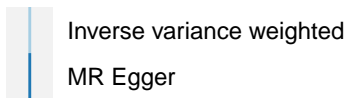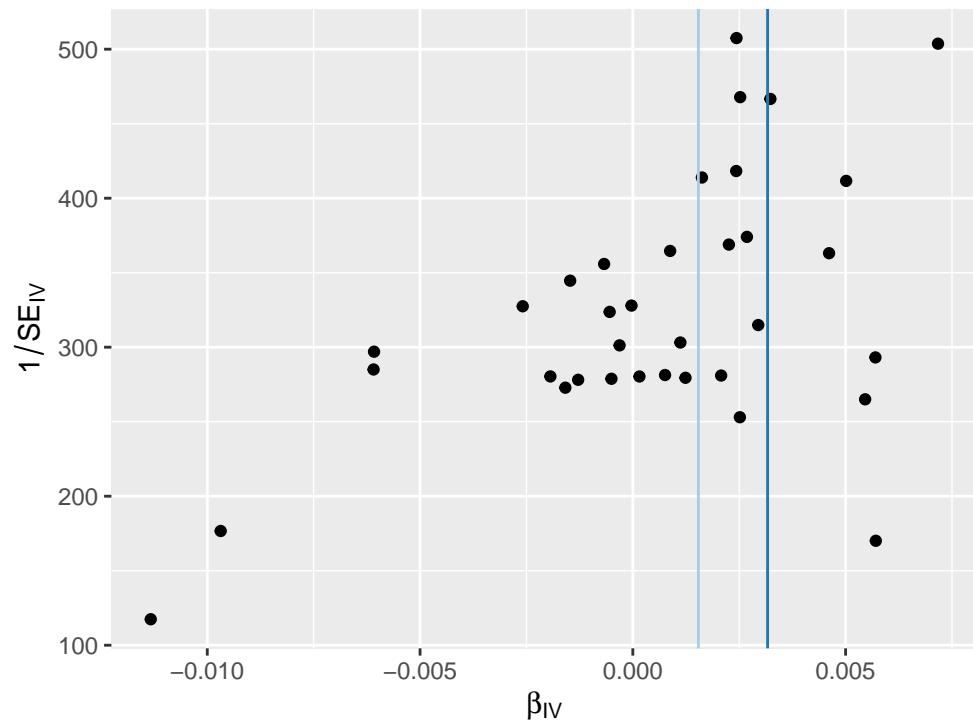

Supplement: Supplementary file 1 [file Data_Sheet_1.PDF]
